# Treatment of condylomata acuminata caused by low-risk human papillomavirus with chloroquine phosphate gel

**DOI:** 10.3389/fmed.2023.1171550

**Published:** 2023-04-28

**Authors:** Xiangling Li, Zhisheng Guan, Qi Liu, Wei Yang, Jie Huang, Manli Yuan, Junlong Yu

**Affiliations:** ^1^Key Laboratory of Basic Research of Traditional Chinese Medicine, Guangxi University of Traditional Chinese Medicine, Nanning, Guangxi, China; ^2^Guangzhou Hybribio Biotechnology Technology Co., Ltd., Guangzhou, Guangdong, China; ^3^Guangdong Lewwin Pharmaceutical Research Institute Co., Ltd, Guangzhou, Guangdong, China; ^4^Department of Dermatology, Shenzhen Third People's Hospital, Shenzhen, Guangdong, China; ^5^School of Basic Medicine, Guangxi University of Traditional Chinese Medicine, Nanning, Guangxi, China

**Keywords:** chloroquine phosphate, human papillomavirus, condylomata acuminata, anogenital warts, p53

## Abstract

**Objective:**

To observe the stability and therapeutic effect of chloroquine phosphate gel on human condylomata acuminata (CA) caused by low-risk human papillomavirus (HPV).

**Methods:**

The appearance, viscosity, pH, chloroquine concentration, deethylchloroquine concentration and content uniformity of chloroquine phosphate gel were examined for 24 months, the gel met the quality standards throughout the 24-month observation. A nude mouse model harboring CA xenografts was used to observe the therapeutic effect of this gel on CA *in vivo*.

**Results:**

After 14 days of gel administration, compared with the control group, the treatment group had significantly smaller warts and significantly reduced DNA copy numbers of HPV6 and HPV11 in the wart tissues. Immunohistochemistry analysis of p53 protein expression in the wart tissues of the treatment group was significantly increased.

**Conclusion:**

Chloroquine phosphate gel was stable and effective against CA, possibly through the promotion of p53 protein expression to induce apoptosis, leading to the involution of warts.

## Introduction

Human papillomavirus (HPV) is a double-stranded DNA virus, and > 200 different subtypes of HPVs have been identified based on the L1 gene sequence (1). Certain low-risk types of HPV, such as HPV6 and HPV11, are associated with condylomata acuminata (CA; anogenital warts) and other types of warts (2). In 90% of cases, the CA is caused by HPV6 and/or HPV11, and in 38% of patients, it is attributed to multiple HPV infections (3). Current treatments for CA in patients infected with low-risk HPVs primarily include self-application of imiquimod, podophyllotoxin and tea polyphenol ointment (4). Medical treatment of CA includes liquid nitrogen freezing, trichloroacetic acid, curettage, laser treatment, surgical resection and ion therapy. However, these treatments have shortcomings (5), such as permanent depigmentation, itching, pain, scarring, bleeding, anal stenosis or incontinence and sepsis (6). It has been shown that it is possible to treat CA by transfecting the human neutrophil peptide 1 gene (HNP1) into CA tissues (7). Another study used 5-aminolevulinic acid photodynamic to treat CA, and the expression intensities of Bcl-2 and surviving varied significantly before and after 5-aminolevulinic acid photodynamic therapy (8). In addition, there are several reports on the treatment of CA using photodynamic therapy alone or in combination with other drugs (9, 10); however, photodynamic monotherapy is expensive and the CA may recur (10). Therefore, it is necessary to continuously explore safe, effective and affordable treatment strategies to manage CA.

As an anti-malarial drug used in clinical practice, chloroquine has seen a decline in use due to the emergence of drug-resistant plasmodium (11). In addition, the application of chloroquine phosphate in the treatment of coronavirus (COVID)-19, at the beginning of 2020, resulted in widespread attention. Several studies on the antiviral effects of chloroquine phosphate have been reported (12, 13); however, the anti-HPV effects of chloroquine phosphate have not been fully elucidated. As early as 1984, oral chloroquine phosphate tablets were used to treat flat warts in China, and the oral cure rate reached 41% (14). In India, hydroxychloroquine was administered to a lady with cutaneous warts twice a day at 200 mg each time for 40 days, and the warts disappeared, with no recurrence after 6 months of follow-up (15). Studies have suggested that the E6 gene of low-risk HPV, such as HPV6 and HPV11, can trap p53 in the cytoplasm (16). p53 is an important tumor suppressor protein that can be activated in response to induce apoptosis, cell cycle arrest, and senescence in cells (17). Kim et al. (18) showed that chloroquine stabilized wild-type p53 expression, and promoted p53-dependent apoptosis and cell cycle arrest in glioma. It activates the p53 pathway and causes apoptosis (18). However, a study on the therapeutic efficacy of chloroquine gel in CA caused by HPV has not been performed previously, to the best of our knowledge. The present study explored the stability of chloroquine phosphate topical gel and determined its effects on a CA-bearing nude mouse model, providing a basis for the novel application of chloroquine phosphate.

## Materials and methods

### Reagents

Different concentrations of chloroquine phosphate topical gels were produced by Guangzhou Hybribio Biotechnology (placebo gel batch no. 151201; chloroquine phosphate gel batch no. 150201). The HPV nucleic acid typing detection kit used in this study was manufactured by Da An Gene Co., Ltd. (Sun Yat-sen University, Guangdong, China; product lot no. 2016002). Other materials used in this study included an anti-p53 antibody (Abcam; cat. no.ab13142; lot no. GR 316086-35), human wart body tissues (Shenzhen Third People’s Hospital, Guangdong, China; all processes met the requirements of the respective Medical Ethics Committee), and BALB/c nu/nu mice [Laboratory animal production license no. SCXK (Hunan) 2013-0004; laboratory animal quality certificate nos. 43,004,700,025,122 and 43,004,700,025,998; approval no. IACUC:IA-PD2015002-01,Guangdong Lewwin Pharmaceutical Research Institute Co., Ltd]. BALB/c nu/nu mice lived in a standard SPF level animal room. The ambient temperature of the animal room was 20 ~ 26°C, the daily temperature difference did not exceed 4°C, the relative humidity was 40 ~ 70%, the number of air changes was greater than or equal to 15 times/h, 12 h of lighting/12 h of darkness alternately, animal illumination 15 ~ 20Lx. The working illumination was not less than 200Lx, the pressure difference between adjacent experimental areas was greater than or equal to 10 Pa, the ammonia concentration in the animal room was not greater than 14 mg/m^3^, and the noise was not greater than 60 dB. Animals eat and drunk freely.

The experimental instruments used in this study included a Leica EG1150H paraffin embedding machine with a EG1150C cold plate, a Leica RM2235 Manual rotary microtome, a Leica ST5020 Multistainer, a Leica DM3000 (ebp100-04-L fluorescence biological microscope), a Leica DFC450 digital microscope camera with a c-mount interface containing a high quality 5 Megapixel CCD sensor with Leica Application Suite software version 4 for image acquisition, a Leica HI1210 water bath for paraffin sections, a Leica HI1220 flattening table (all from Lecia Microsystems GmbH), a high-performance liquid phase detector (Agilent 1260 Infinity Multi-detector; Agilent Technologies, Inc.) pH meter (Mettler, FE28) and a viscometer (Shanghai Fangrui Instrument, Co., Ltd., NDJ-8S).

### Assessment of the stability of chloroquine phosphate gel

According to the Pharmacopoeia of the People’s Republic of China (19), the long-term stability test conditions were set at a temperature of 25 ± 2°C and relative humidity (RH) 60 ± 10%, and the test time points were 0, 3, 6, 9, 12, 18 and 24 months. The test indicators included appearance, viscosity, pH, chloroquine concentration, content uniformity, deethylchloroquine concentration and microbial limits after 12 and 24 months. The chloroquine and deethylchloroquine concentrations, as well as content uniformity were measured using a high-performance liquid phase detector. Follow the Pharmacopoeia of the People’s Republic of China (20), plate culture was used to detect the common pathological bacteria, including *Staphylococcus aureus*, *Candida albicans*, *Escherichia coli* and *Pseudomonas aeruginosa* to ensure product quality.

### Condylomata acuminata animal modeling and drug testing

In vivo xenograft model of CA. A total of 10 nude mice, half meal and half female, 19.3 ± 2.5 g were used in the present study. Because 20–240 mg/kg propofol may not be a significant modifiable factor for neuroinflammation and cognitive impairment caused by surgery under general anesthesia (21, 22), based on the time of surgery and veterinary assessment, 40 mg/kg 4% propofol was used for the animals were anesthetized by tail vein injection of 40 mg/kg 4% propofol before the modeling. The outpatient surgical resections of anogenital warts with confirmed HPV infection were directly inoculated under the skin of the nude mice to observe their growth. Briefly, the clinical specimens were rinsed with 100 U/ml normal saline containing both penicillin and streptomycin (Beyotime Biotechnology), followed by trimming and scraping off the subcutaneous tissues. The specimen blocks were then cut into tissue blocks of ~ 0.8 × 0.8 × 0.5 cm^3^ in size. After disinfecting the skin on the back of the neck of each nude mouse, a scalpel was used to cut individual holes at an inclination angle of ~ 30° to reach the muscle layer. Guided forceps were used to open each tiny hole, and a wart tissue block was inserted into the bottom of each hole, followed by suturing of the wound to fix the wart tissue. The entire procedure was performed strictly under aseptic conditions. The wart tissue blocks were kept moist during the operation. After the operation, the operational area was covered by an oil gauze and then a sterile bandage. The bandage was opened ~ 24 h after the operation, and then 0.2 ml Matrigel^™^ (Corning) was injected into the base the following day. H&E staining and detection of HPV11 and HPV6 were used to determine the success of the modeling.

### Formal test

A total of 40 nude mice, half meal and half female,19.3 ± 2.5 g were selected for CA modeling; 5–6 days after wart implantation, the nude mice with good inoculation sites were selected and randomly divided into four groups: Control group (placebo gel treatment), and 2.5 (mild-dose), 5 (medium-dose) and 10 mg/cm^2^ (high-dose) chloroquine phosphate gel-treatment groups (6 mice per group, 1:1 male-to-female ratio). These dosages are, respectively, equivalent to 0.25, 0.5 and 1X the clinical adult dosage. The corresponding topical treatments were given to the animals via skin smearing (administration area of ~ 2 × 2 cm on the wart inoculation surface) once a day to allow the corresponding treatments to contact the skin for 6 h, followed by removal of the drug from the skin. The entire treatment course lasted for 14 consecutive days. The animals in each group were clinically monitored daily during the drug administration period. The growth of wart xenografts was observed daily, and the body weight of each nude mouse was measured every 3 days. On the day after the final administration, the animals were weighed and euthanized by cervical dislocation to harvest and weigh the wart xenografts, and to calculate the inhibition rate of wart xenografts with reference to the evaluation index of tumor growth rate.

### Human papillomavirus gene detection in the wart tissues

The wart xenografts were used to detect HPV6 and HPV11 DNA using a fluorescence-based quantitative PCR in accordance with the manufacturer’s instructions of the detection kit (Da An Gene Co., Ltd.). Steps were as follows: (1) DNA extraction: Took about 50 mg of tissue, add 1 ml of sterile saline and grind it into a homogenizer with a homogenizer, transferred it to a 1.5 ml centrifuge tube, and centrifuged at 12000 rpm for 5 min. Remove the supernatant, add 1 ml of sterile physiological saline to the pellet, shake and mix thoroughly, and centrifuged at 12000 rpm for 5 min. Remove the supernatant, added 50 μL DNA extraction solution to the pellet and mix well. Incubated at 100°C for 10 min, and then centrifuge at 12000 rpm for 5 min for later use. (2) HPV6,11 positive quality control product (1 × 10^6^copies/μL): Centrifuge the positive quality control product at 6,000 rpm for a few seconds and mark it as 1 × 10^6^. Took 4 sterilized 0.5 ml centrifuge tubes and added 45 ml negative control products respectively, then marked as 1 × 10^5^ ~ 1 × 10^2^. Took 5 μL of the 1 × 10^6^ positive quality control product to the Ix10^5^ tube and mixed it repeatedly with a sampler, then changed the tip, and diluted it to 1 × 10^2^ in this way. Took 40 μL for each gradient, and added 40 μL DNA extraction solution to mix well. Incubated at 100°C for 10 min, and centrifuged at 12000 rpm for 5 min. (3) PCR amplification: Take 2 μl of the processed sample and quantitative quality control standard for simultaneous amplification. PCR amplification conditions are: 93°C 2 min pre-denaturation, then 93°C 45 s, 55°C 60s for 10 cycles, then 93°C 30s, 55°C 45 s for 30 cycles. (4) The result was automatically analyzed and calculated by the computer to give the copy number. The results with amplification curves not exhibiting a sigmoid shape or with a Ct ≥ 30 were considered as an indication that the total contents of HPV6 and HPV11 DNA in the warts were below the detection limit. If the amplification curve exhibited a sigmoidal shape and a Ct < 30, and if the total gene copy content (C) was < 5.0 × 10^2^, the total content of HPV6 and HPV11 DNA was < 5.0 × 10^2^ gene copies; if C was > 5.0 × 10^2^ and < 5 × 10^8^, the total content of HPV6 and HPV11 DNA was C gene copies; if C was > 5 × 10^8^, the total content of HPV6 and HPV11 DNA was > 5 × 10^8^ gene copies.

### Histopathological examination of wart xenografts

A total of 14 days after topical drug administration at room temperature, the wart xenografts were harvested, postfixed with 10% neutral formalin for 24 h, paraffin-embedded and sectioned into 4-μm thick slices, followed by staining with H&E at room temperature for 5 min with hematoxylin and 4 min with eosin, washed, then to observe the histopathology under a light microscope(× 400).

### p53 protein expression in the wart xenografts

A total of 14 days after topical drug administration, the wart xenografts were harvested, postfixed, paraffin-embedded and sectioned into 4-μm thick slices, followed by immunohistochemistry analysis. Anti-rabbit p53 polyclonal antibodies (Abcam; cat. no.ab13142; lot no. GR 316086-35) were used to determine total p53 protein expression according to the manufacturer’s instructions, incubation in 37°C constant temperature water bath for 2 h, and observed under a light microscope(× 200, × 400).

### Statistical analysis

SPSS version 25.0 (IBM Corp.) was used for statistical analysis. Data are presented as the mean ± SD. One-way ANOVA was used for comparison of means between multiple groups, and LSD test was used for comparison between two groups. *p* < 0.05 was considered to indicate a statistically significant difference.

## Results

### Stability of the chloroquine phosphate gel

The chloroquine phosphate gel was a transparent or light yellow semi-solid gel with a pH value between 4 and 6 and a viscosity of 40,000–80,000 mPa/s. The labeled amount of chloroquine phosphate in the gel was between 90 and 110%. The long-term stability of chloroquine phosphate gel was assessed. The changes in the various indicators of the product between 0 and 24 months were as follows: After the chloroquine phosphate gel was stored for 24 months under long-term stable conditions, the gel became darker in color, the viscosity changed from 77,297.00 mPa/s to 72,691.89 mPa/s, content slightly decreased from 1.0145 to 0.9617 g, and the pH value increased from 4.71 to 4.82 (Table 1). However, these changes were all within the test error range, indicating that the gel product was stable after 24 months of storage.

**Table 1:** Changes in the long-term stability of chloroquine phosphate gel from 0 to 24 months.

Indicator	0 month	3-months	6-months	9-months	12-months	24-months	Change/%
Trait	Light yellow	Light yellow	Light yellow	Light yellow	Light yellow	yellow	Darkening of color
pH value	4.71	4.84	4.95	4.92	4.82	4.82	2.34
Viscosity (mpa/s)	77297.00	74073.93	62514.27	74644.50	64912.44	72691.89	−5.96
Content uniformity (%)	96.63	102.73	100.37	100.50	101.17	95.94	−0.71
Content (g)	1.0145	1.0134	1.0474	1.0182	1.0275	0.9617	−5.20

### Confirmation of establishment of the CA model

**Figure 1:**
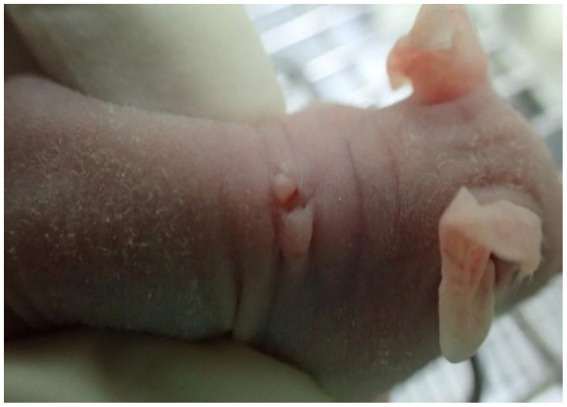
A representative image of a nude mouse after the inoculation of CA tissue. After CA tissue inoculation in the nude mouse, the skin around the operational area was dry with no discharge, and the wart was slightly raised above the skin surface, resembling the skin of a nude mouse, with a hard texture when touched. CA, condylomata acuminata.

**Figure 2:**
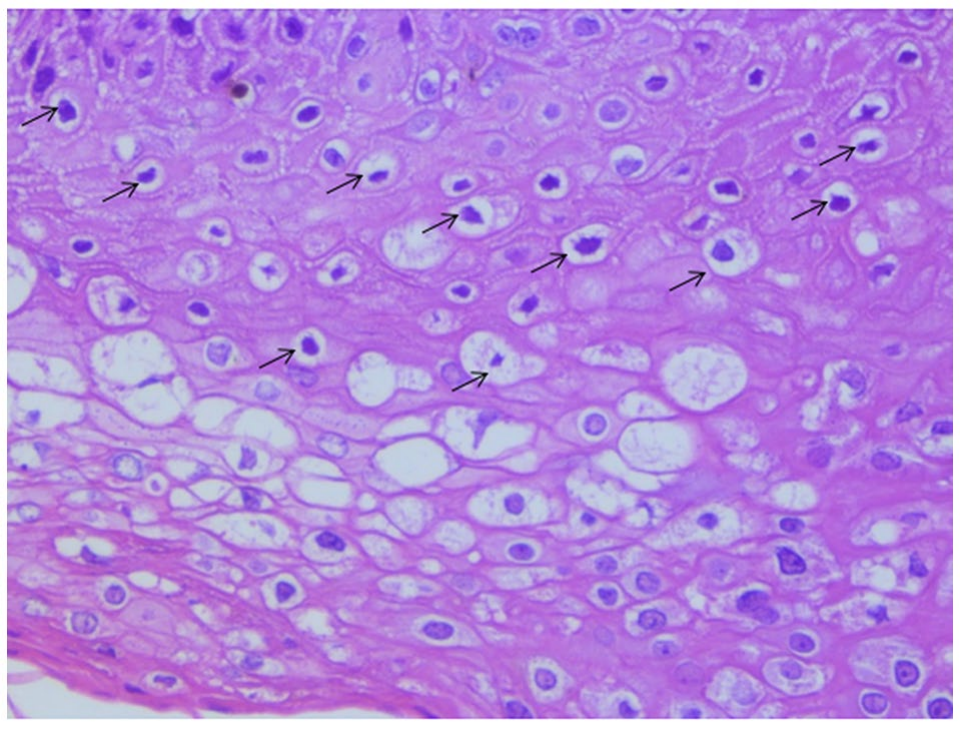
Representative image of H&E staining of the wart xenograft. Magnification, × 400. Koilocytes were important indicators for condyloma acuminatum. As arrows shown in this figure, koilocytes were observed in the granular layer and the upper part of the spinous layer. That means the CA model was successful to use in the next experiments.

The wart-bearing nude mice were healthy after wart inoculation and exhibited normal food intake. The skin around the operational area was dry with no discharge. A total of 1 week after the operation, the wound edges were aligned neatly without cracks or varying thickness. In addition, the scab had fallen off, and new warts had grown. The warts were slightly raised above the skin surface and looked similar to the skin of a nude mouse, with a hard texture when touched (Figure 1). Animals with a Ct = 17.47 in the skin HPV test were positive for HPV infection. Because koilocytes were identified in all the HPV positive cases, they were the important indicators for condyloma acuminatum (23). In Figure 2, the general and histopathological results 14 days after the operation were similar to the characteristics of CA with a significantly increased number of koilocytes. These results indicated that the pathological and biological characteristics of the wart-bearing nude mice under the experimental conditions were similar to those of human CA, suggesting the successful establishment of the model in the animals.

### Effects of chloroquine phosphate on the CA animal model

**Figure 3:**
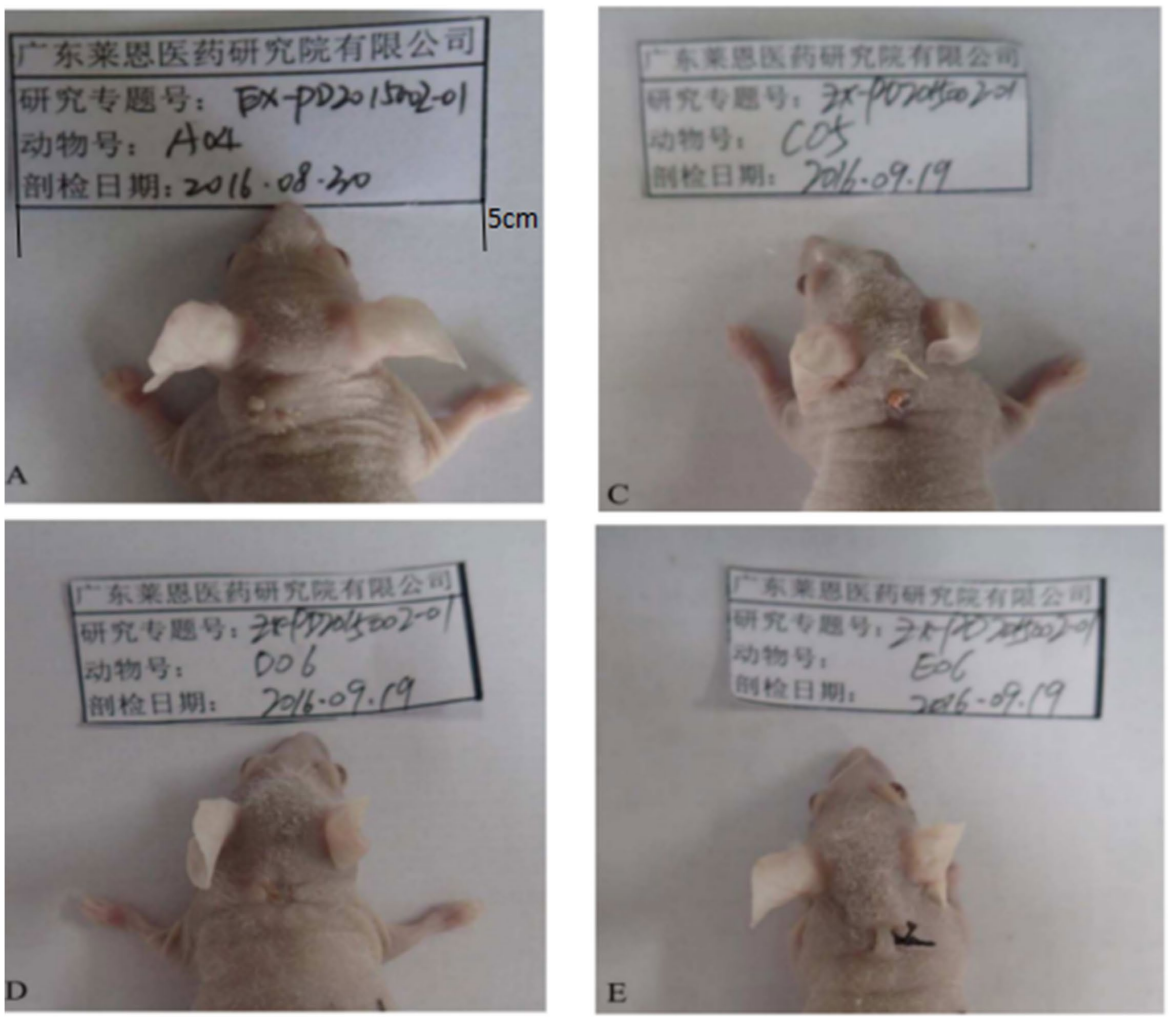
Changes of transplanted warts after 14 days of administration. **A** is the control group. From **A**, it can be seen that the warts were still growing and new small warts were growing out. **C** was the low-dose group. From **C**, the growth of the wart body was inhibited, and the wart body was obviously shrinking and smaller. **D** was the medium-dose group. From **D**, the wart body had basically fallen off. **E** was the high-dose group. In **E**, compared with **A**, the wart was smaller, no new warts grow out.

The experimental results indicated no obvious abnormalities in body weight of the animals in the different groups of mice during the experiments (Table 2). After topical drug administration, the warts were weighed, and the weight of the warts in each chloroquine gel treatment group was significantly reduced (*p* < 0.01; Table 2; Figure 3).

**Table 2:** Effect of chloroquine phosphate gel on body weight and wart weight of condyloma acuminatum-bearing nude mouse model (*n* = 6 each group).

Group	Initial bodyweight (g)	Last bodyweight (g)	Wart weight (g)	Wart growth inhibition rate (%)
Control group	18.8 ± 2.1	23.1 ± 2.3	0.231 ± 0.056	
Low-dose group	19.8 ± 2.5	20.9 ± 1.8	0.115 ± 0.065^**^	50.2
Medium-dose group	19.1 ± 2.9	22.0 ± 3.1	0.111 ± 0.026^**^	51.9
High-dose group	19.4 ± 2.7	21.2 ± 3.2	0.120 ± 0.051^**^	48.1

^*^p < 0.05, ^**^p < 0.01, compared with the model control group.

### Human papillomavirus content in the wart xenografts of the CA model

Determination of HPV6 and HPV11 DNA content in the wart xenografts using fluorescence-based quantitative PCR showed sigmoidal shaped PCR amplification curves in the positive quality control, strong positive quality control and positive quantitative standards. The measured quantitative reference values of quality control products were all within the range of gene copy, with Ct < 27, standard curve *R*^2^ = 0.998, and 0.97 ≤ *R*^2^ ≤ 1, indicating that the test system was stable and the results were reliable (Table 3). Compared with the model control group, the HPV6 and HPV11 DNA contents of the wart xenografts of the low-dose, medium-dose and high-dose chloroquine phosphate gel-treatment groups were all significantly lower than the control group (*p* < 0.05).

**Table 3:** Effect of chloroquine phosphate gel on HPV6 and HPV11 Ct values and DNA contents of warts in the condyloma acuminatum-bearing nude mouse model (*n* = 6 each group).

Group	Ct value	DNA (copies/mL)
Control group	15.8 ± 5.6	3.27E9 ± 2.86E9
Low-dose gel group	20.7 ± 1.4^*^	2.34E7 ± 1.40E7^**^
Medium-dose group	22.9 ± 1.7^**^	6.67E6 ± 5.90E6^**^
High-dose group	20.1 ± 3.3^*^	2.37E8 ± 5.08E8^**^

^*^p < 0.05, ^**^p < 0.01, compared with the model control group.

### Histopathological examination of the wart xenografts

Warts were observed in all animals in the model control group, and they exhibited an intact epithelial cell structure and koilocytes in certain areas. In the low-dose group, warts were observed in all animals; 2 out of 6 animals showed locally degenerated and necrotic epithelial cells and a few koilocytes in the warts, and the other 4 out of 6 animals exhibited complete epithelial cell structure and koilocytes in certain areas. In the medium-dose group, warts were observed in all animals; 3 out of 6 animals showed locally degenerated and necrotic epithelial cells and a small number of koilocytes in the warts, and the other 3 out of the 6 animals exhibited complete epithelial cell structure and koilocytes in the warts. In the high-dose group, warts were observed in all animals; 4 out of 6 animals showed locally degenerated and necrotic epithelial cells in the warts, and the remaining 2 out of 6 animals showed complete epithelial cell structure and koilocytes locally in the warts (Table 4).

**Table 4:** Results of histopathological examination of wart xenografts (*n* = 6 each group).

Histopathological phenomena	Number of each group			
	Control group	Low-dose group	Medium-dose group	High-dose group
Intact epithelial tissue, hollow cells	6	4	3	2
Epithelial local degeneration and necrosis, a few hollow cells	0	2	3	4

**Figure 4:**
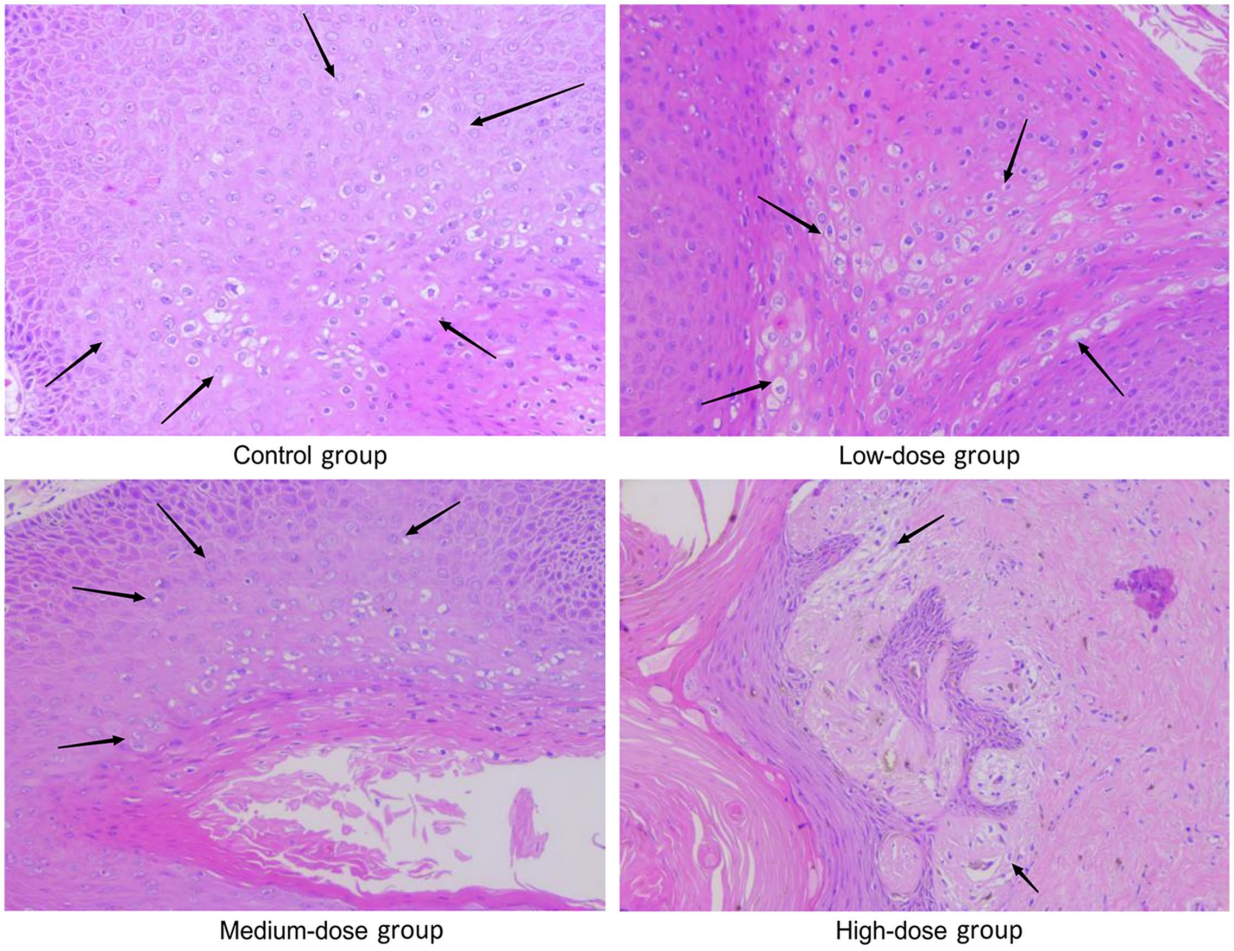
Representative images of H&E staining of the wart xenografts in the different groups of nude mice. Magnification, × 200. In the model control group, as arrows shown koilocytes around the nucleus of local epithelial cells, and the nucleolus of the nucleus were observed; in the low-dose and medium-dose groups, as arrows shown, koilocytes around the nucleus of local epithelial cells, and the nucleolus of the nucleus were observed. In the high-dose chloroquine phosphate gel-treatment group, hyperkeratosis of the epidermal cells of the wart body, squamous epithelium under the epidermis, local epithelial cell degeneration and necrosis was visible, and a small quantity of koilocytes could be seen locally, just as arrows shown.

As shown in Figure 4, koilocytes around the nucleus of the local epithelial cells and the nucleoli in the warts were observed in the control group, the low-dose group and the medium-dose group. In the high-dose group, hyperkeratosis was observed in the epithelial cells of the warts, and squamous epithelium was observed under the epidermis, showing local degeneration and necrosis in the epithelial cells, with a small number of koilocytes in the warts. From the results in Table 4 and Figure 4, it can be seen that, with the increase in the dose of chloroquine phosphate gel, the probability of necrosis of the wart epithelial tissue increased, indicating that chloroquine phosphate gel was irritating to the skin to a certain degree, and as the dose increases, the irritation becomes greater.

### p53 protein expression in the wart xenografts

**Figure 5:**
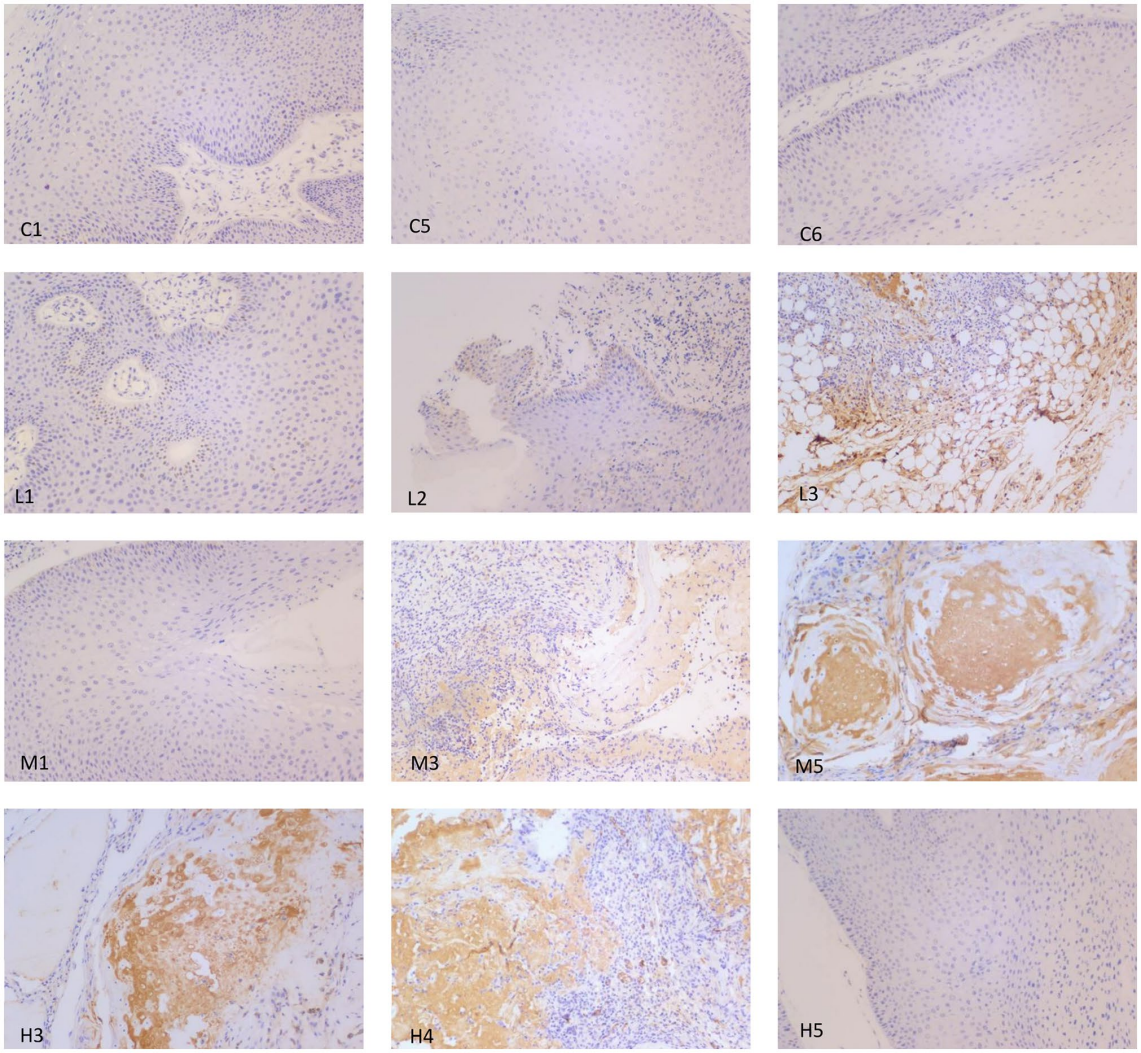
Representative images of p53 immunohistochemistry analysis in the different groups of nude mice. Magnification, × 200. In the control group, tissue samples from mice nos. 1, 3 and 5 (C1, C5 and C6, respectively) were selected for p53 staining, and the results showed that there was no visible expression of p53 in the three samples. In the low-dose group, tissue samples from mice nos. 1, 2 and 3 (L1, L2 and L3, respectively) were used for p53 staining. Only a small number of p53-positive cells were expressed per section. In the medium-dose group, tissue samples from mice nos. 1, 3 and 5 were used for p53 staining. Only a small number of p53-positive cells were observed in M1. M3 and M5 showed strong positive expression of p53. In the high-dose group, tissue samples for mice nos. 3, 4 and 5 were selected for p53 staining. A section of H3 exhibited a small amount of p53-positive cell expression, and H4 and H5 showed strong positive expression of p53, as well as cell apoptosis and tissue incompleteness.

**Figure 6:**
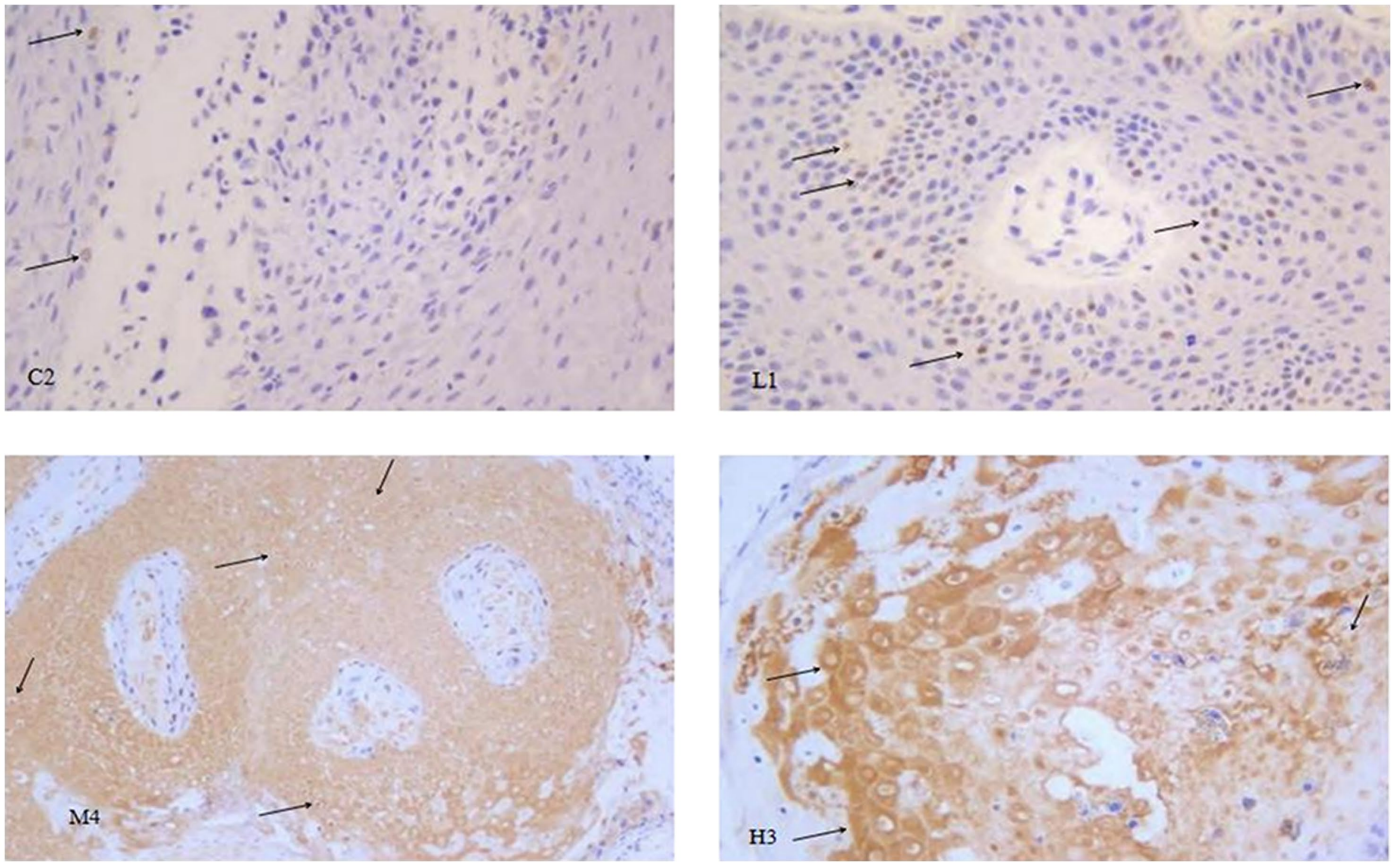
Representative images of p53 immunohistochemistry in the different groups of nude mice. Magnification, × 400. Through careful observation, in the control group C2, koilocytes cells were clearly visible, and there were very few p53-positive cells, as indicated by the arrow in the figure. The number of p53-positive cells in the low-dose group L1 was notably higher than that in the control group. The wart tissues in the medium-dose group M4 were strongly p53-positive. In the high-dose group H3, in addition to the strong p53 positive staining, cell necrosis was also clearly observed.

Immunohistochemistry was used to analyze the expression of p53 protein in the wart xenograft tissues in the different groups of nude mice. As shown in Figure 5, in the three animal wart tissue sections of the blank control group, there were almost no p53-positive cells. In the low-dose group, there were only a few p53-positive cells in each animal tissue section. In the medium-dose group, 2 of the 3 mouse tissue sections were strongly positive, and 1 film had only a few positive cells. The high-dose group results were the same as that observed in the medium-dose group. Further in-depth comparison of p53-positive cells in each group showed that in the control and low-dose groups, p53-positive cells were relatively scattered, whereas in the medium-dose and high-dose groups, strong p53-positive expression was visible in the entire tissue. In the high-dose group, cell apoptosis and tissue incompleteness were notably visible (Figure 6). This is consistent with the results observed in the HE staining. Although certain tissues in the medium-dose and high-dose groups were not positive for p53, this may be related to the location and section of the tissue.

Compared with the model control group, the positive staining of p53 in the warts was significantly enhanced as the dose of chloroquine phosphate increased, showing a dose-dependent relationship between the p53 protein expression and chloroquine phosphate.

## Discussion

Chloroquine phosphate was discovered in the 1960s (24), and has been primarily used in the form of tablets to treat malaria (25); however, there are also studies assessing the pharmacology and toxicology of chloroquine phosphate injection as a treatment for malaria (26, 27). Recently, chloroquine phosphate tablets were assessed as a potential treatment for COVID-19 in patients based on the anti-SARS effect of chloroquine, although no clinical trials have shown its benefits in the treatment of COVID-19 (28). The adverse reactions of high-dose oral chloroquine phosphate tablets have been a central focus of concern and show the need for further treatment options. Based on the antibacterial and antiviral activity of chloroquine phosphate (29, 30), as well as its water-soluble characteristics, a new formulation, namely a chloroquine phosphate gel, was produced, and its long-term stability according to the requirements of the Pharmacopoeia of the People’s Republic of China (2015 Edition) was assessed. The results of the stability test showed that the chloroquine phosphate gel exhibited a low degree of change in appearance, viscosity, pH, chloroquine concentration, content uniformity and deethylchloroquine concentration after 24 months of storage, indicating that the product was stable. This product was similar to the chloroquine phosphate anti-HIV gel made by Brouwers et al. (31). However, the chloroquine phosphate water-soluble gel developed for the present study was simpler, as the aqueous solution of chloroquine phosphate itself was acidic, thus not requiring pH adjustment.

Condylomata acuminata is a relatively common sexually transmitted disease caused by low-risk HPV; ~ 1% of sexually active individuals will get the disease (4). While it is not usually a serious problem, it can cause emotional distress to a patient and even physicians, due to its tendency for recurrence (6). To assess HPV-induced CA, specifically by a low-risk HPV infection, an immuno-compromised nude mouse model received a wart xenograft to generate a human CA-like animal model, and was used to observe the effectiveness of the chloroquine phosphate gel. After confirming the successful modeling in the animals, the gels of different concentrations were applied topically for 14 days on the wart. Compared with the model control group, the three doses of chloroquine phosphate gel (low, medium and high) inhibited the growth of warts in a dose-dependent manner, and the number of HPV DNA copies was reduced, indicating that chloroquine phosphate inhibited the replication of HPV DNA to a certain extent. In the follow-up test of the mechanism of gel action, a study on the effect of chloroquine phosphate gel on condyloma acuminatum caused by different subtypes of HPV would be conducted. At present, HACAT-HPV11 cell line was constructed to conduct *in vitro* experiments to further study the mechanism of chloroquine phosphate on HPV.

According to the H&E-staining results, the number of necrotic epithelial cells in the warts of the chloroquine phosphate gel-treatment group increased as the dose of chloroquine phosphate increased. Based on p53 immunohistochemistry analysis, it was shown that the expression of p53 protein increased as the dose of chloroquine phosphate increased. The experimental results indicated that chloroquine phosphate gel may promote the expression of p53 protein, resulting in apoptosis of the wart cells, thus eliminating the warts infected with HPV.

p53 is an endogenous tumor suppressor protein and is referred to as ‘the guardian of the genome’ due to its ability to guide DNA repair or induce apoptosis (17). p53 effectively inhibits cell proliferation, temporarily or permanently, in response to various types of cell stresses to activate the cell death program or prevent proliferation (18). The HPVE6 protein binds to p53 and degrades or inactivates p53, thereby inhibiting the stabilization of p53. p53 stabilization affects HPVE7 expression (32). This allows the virus to ‘control’ the cell and reproduce copies of itself. Since the HPV E6 protein degrades p53, it allows the infected cell to replicate itself and prevents apoptosis (32). In the present study, chloroquine phosphate gel increased the expression of p53 protein and a high dose resulted in the necrosis of the epithelial cells of the CA, possibly due to the expression of the p53 protein. However, chloroquine can cause DNA damage, and the magnitude of DNA damage increases as a function of the chloroquine concentration administered (33). DNA damage also leads to an increase in cellular p53 levels (17). Further experimental verification is thus required to determine how chloroquine phosphate promoted the expression of p53, and whether it can be achieved by inhibiting the HPV virus E6, via DNA damage or both.

In conclusion, the chloroquine phosphate gel product developed was stable for 24 months, with an inhibitory effect on nude mice bearing wart xenografts. It was also shown that the underlying mechanism of chloroquine phosphate gel involved increased p53 protein expression in the CA when administered topically. This chloroquine phosphate gel product is currently undergoing clinical trials.

## Data availability statement

The original contributions presented in the study are included in the article/Supplementary material, further inquiries can be directed to the corresponding author.

## Ethics statement

The animal study was reviewed and approved by Guangdong Lewwin Pharmaceutical Research Institute Co., Ltd. [Laboratory animal production license no. SCXK (Hunan) 2013-0004; laboratory animal quality certificate nos. 43004700025122 and 43004700025998; approval no. IACUC:IA-PD2015002-01]. Written informed consent was obtained from the owners for the participation of their animals in this study.

## Author contributions

ZG and XL assisted in the development of the product, and reviewed and edited the manuscript. QL and MY designed and performed the experiments, and wrote, reviewed and edited the manuscript. WY and JY established the *in vivo* model, analyzed the data, and reviewed and edited the manuscript. JH provided human wart tissue. ZG, JH, and XL confirmed the authenticity of all the raw data. JY and XL designed and planned the study, performed the experiments, and wrote, reviewed and edited the manuscript. All authors contributed to the article and approved the submitted version.

## Funding

This work was supported by Guangxi University of Traditional Chinese Medicine Doctoral Program: 2020BS011 and 2021BS019.

## Conflict of interest

ZG, QL, and MY were employed by the company Guangzhou Hybribio Biotechnology Technology Co., Ltd. WY was employed by the company Guangdong Lewwin Pharmaceutical Research Institute Co., Ltd.

The remaining authors declare that the research was conducted in the absence of any commercial or financial relationships that could be construed as a potential conflict of interest.

## Publisher’s note

All claims expressed in this article are solely those of the authors and do not necessarily represent those of their affiliated organizations, or those of the publisher, the editors and the reviewers. Any product that may be evaluated in this article, or claim that may be made by its manufacturer, is not guaranteed or endorsed by the publisher.
